# Valid and efficient manual estimates of intracranial volume from magnetic resonance images

**DOI:** 10.1186/s12880-015-0045-4

**Published:** 2015-02-18

**Authors:** Niklas Klasson, Erik Olsson, Mats Rudemo, Carl Eckerström, Helge Malmgren, Anders Wallin

**Affiliations:** Institute of Psychiatry and Neurochemistry, Department of Neuroscience and physiology, The Sahlgrenska Academy, University of Gothenburg, Box 430, SE-405 30 Göteborg, Sweden; Department of Mathematical Sciences, Chalmers University of Technology and University of Gothenburg, SE-412 96 Göteborg, Sweden; Department of Philosophy, Linguistics and Theory of Science, University of Gothenburg, Box 200, SE-405 30 Göteborg, Sweden

**Keywords:** Intracranial volume, Manual estimation, Magnetic resonance imaging, Validity

## Abstract

**Background:**

Manual segmentations of the whole intracranial vault in high-resolution magnetic resonance images are often regarded as very time-consuming. Therefore it is common to only segment a few linearly spaced intracranial areas to estimate the whole volume. The purpose of the present study was to evaluate how the validity of intracranial volume estimates is affected by the chosen interpolation method, orientation of the intracranial areas and the linear spacing between them.

**Methods:**

Intracranial volumes were manually segmented on 62 participants from the Gothenburg MCI study using 1.5 T, T_1_-weighted magnetic resonance images. Estimates of the intracranial volumes were then derived using subsamples of linearly spaced coronal, sagittal or transversal intracranial areas from the same volumes. The subsamples of intracranial areas were interpolated into volume estimates by three different interpolation methods. The linear spacing between the intracranial areas ranged from 2 to 50 mm and the validity of the estimates was determined by comparison with the entire intracranial volumes.

**Results:**

A progressive decrease in intra-class correlation and an increase in percentage error could be seen with increased linear spacing between intracranial areas. With small linear spacing (≤15 mm), orientation of the intracranial areas and interpolation method had negligible effects on the validity. With larger linear spacing, the best validity was achieved using cubic spline interpolation with either coronal or sagittal intracranial areas. Even at a linear spacing of 50 mm, cubic spline interpolation on either coronal or sagittal intracranial areas had a mean absolute agreement intra-class correlation with the entire intracranial volumes above 0.97.

**Conclusion:**

Cubic spline interpolation in combination with linearly spaced sagittal or coronal intracranial areas overall resulted in the most valid and robust estimates of intracranial volume. Using this method, valid ICV estimates could be obtained in less than five minutes per patient.

## Background

Intracranial volume (ICV) estimated by magnetic resonance images (MRI) is often used as a proxy variable for premorbid brain volume in brain volumetric studies. ICV normalization has been used to reduce interindividual variance in both whole brain volume [1] and regional brain volumes, e.g. hippocampus [2,3]. To save time, estimates of ICVs are commonly calculated by the sum of linearly spaced intracranial areas (ICA) multiplied by the distance between those areas. Eritaia et al. evaluated how the validity of such estimates decreases as the linear spacing increases using sagittal ICAs [4]. They found that the validity not only decreased with increased linear spacing, but also grew more uncertain due to an oscillation in the functions reported. To avoid this uncertainty it has been common to use small linear spacings of no more than 10 mm between sagittal ICAs e.g. [5,6]. In these cases the validity of the estimates should be high, with an intra-class correlation of about 0.999 [4]. However, the adoption of the method is often hard to appraise as it tends to be sparsely reported, e.g. leaving out choice of linear spacing [7-10].

**Figure 1:**
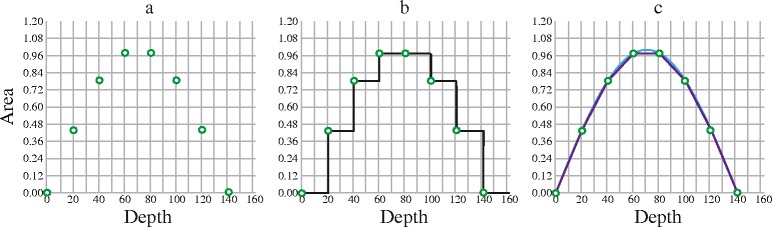
**Estimating a volume by linearly spaced areas. a)** The green dots illustrate known, linearly spaced, areas from an unknown volume. The Y-axis gives the size of these areas and the X-axis at which depth, in the unknown volume, they were measured. **b)** By multiplying each known area by the linear spacing, the lost areas are approximated (black border), a piecewise constant interpolation is made. The area under the curve is the estimate of the unknown volume. **c)** The lost areas approximated by two piecewise polynomial interpolations, a piecewise linear interpolation (purple graph) and a cubic spline interpolation (blue graph).

Information about the shape and volume of an intracranial vault is lost when only a few ICAs are segmented. From the known ICAs this lost information can be approximated by different means. When multiplying the sum of ICAs by the linear spacing between them, a piecewise constant interpolation is made (Figure 1,a-b). This is the simplest form of interpolation method, and the oscillation seen in the study by Eritaia et al. reflects its irregular performance [4]. If we also, besides the linear spacing, note the order of the known ICAs, better approximations of the lost ICAs may be achieved. In this case we can assume that ICAs between two known ICAs follow a linear function or other polynomial functions. These assumptions can be implemented using so called piecewise polynomial interpolations (Figure 1c). The possibility of achieving more valid ICV estimates by using piecewise polynomial interpolation seems not to have been investigated before. Further, the validity of ICV estimates using linearly spaced ICAs in coronal or transversal orientation is still unknown.

While the method evaluated by Eritaia et al. [4] has been used both to evaluate other ICV estimation methods [11,12] and to correct for differences in premorbid brain volume [5,6], the original study itself has not been validated. The aim of the present study was therefore to 1) replicate the study by Eritaia et al., in a different sample, in order to validate their findings, 2) evaluate the validity of ICV estimates from coronal and transversal ICAs, and 3) evaluate different interpolation methods that could improve the validity of the ICV estimates.

## Methods

### Participants

The present study is part of the Gothenburg MCI (mild cognitive impairment) study from which a subsample of 38 patients and 32 controls with 1.5 T MRI scans was included. The patients, who were referred to the memory clinic in Mölndal with subjective or objective cognitive impairment, were first included in the Gothenburg MCI study if they did not fulfill any of the exclusion criteria. Exclusion criteria were severe somatic or psychiatric disorder, alcohol or substance abuse, confusion caused by drugs, and pseudodementia. Patients were classified into one of four stages using the global deterioration scale (GDS) for assessment of primary degenerative dementia [13], where stage 1 means no cognitive decline, stage 2 is subjective cognitive impairment, stage 3 is mild cognitive impairment and stage 4 indicates dementia. Controls were mainly recruited from organizations for senior citizens and were excluded if they had any sign of cognitive impairment. To participate in the Gothenburg MCI study both patients and controls had to give their written informed consent. The Gothenburg MCI study has previously been described [14] and has been approved by the ethics committee of Gothenburg University (diary number: L091-99, 1999; T479-11, 2011).

Eight of the 70 participants in the present substudy were excluded because parts of the intracranial vault had not been covered in the MRI scans. Of the excluded subjects two (25 percent) were women. Two had GDS 4, three had GDS 2–3 and three were controls. Of the remaining 62 participants, 39 (63 percent) were women. Twenty-five had GDS 4, eight had GDS 2–3 and 29 were controls. The excluded and the remaining subjects did not differ significantly with respect to age, education or MMSE, but differed significantly regarding gender. The complete demographics of the remaining participants can be viewed in Table 1.

**Table 1:** **Study demographics for the remaining participants**

**Group belonging**	**N**	**Gender** **(m/** **f)**	**Age**	**Education**	**MMSE**
Controls	29	8/21	66.4 ± 7.5	11.5 (7.0, 15.0)	30 (27, 30)
Patients, GDS 2-3	8	4/4	66.7 ± 8.2	12.0 (6.5, 20)	28.5 (26.0, 29.0)
Patients, GDS 4	25	11/14	65.5 ± 8.8	10.0 (6.0, 23.0)	25 (16, 30)

Study demographics for the remaining controls, patients with a global deterioration scale (GDS) score of 2–3 and patients with GDS score of 4. N gives the number of participants and the gender ratio is given as males (m) per females (f). Age is described with means and standard deviations while education and mini-mental state examination (MMSE) are described with medians with minimum and maximum values enclosed in parentheses.

### MRI acquisition

Coronal, T_1_-weighted, 3D IR/GR (inversion recovery/gradient echo) MRI scans, obtained from a 1.5 T Siemens Symphony scanner, were used for the ICV segmentations. The coronal plane was aligned perpendicular to the longitudinal axis of hippocampus during the MRI examinations. All MRI examinations were performed at Sahlgrenska University Hospital, Mölndal radiology department. The acquisition parameters were: echo time = 2.38 ms, field of view = 250 x 203 mm, flip angle = 15°, inversion time = 820 ms, matrix size = 512 × 416, pixel spacing = 0.49 × 0.49 mm, repetition time = 1610 ms, slice thickness = 1 mm, bit depth = 12.

### Image analysis

The ICV segmentations were performed manually using a MacBook Pro 13” with an Intel Core i7 processor, an Intel HD Graphics 3000 graphic card, and an external Wacom DTU-2231 interactive pen display with about 102 pixels per inch resolution and a system gamma of 1.8. The segmentations were acquired using a custom built software (MIST) developed by N. Klasson for manual segmentation of MRI volumes.

The MRI scans were reformatted into 1 mm cubic voxels using linear interpolation, but no realignment of the images was done to correct for head tilt. A simple method was used to adjust the MRI scans to the same intensity level. Essentially, the mean intensity of each MRI scan was adjusted in brightness so that it matched the pre-adjustment mean intensity of all MRI scans. However, if the amount of background in a scan is large (e.g. due to a small head), its mean intensity tends to be lower compared to a scan with less background (e.g. due to a large head). The mean intensity would in this case reflect the amount of background rather than the brightness of the MRI scan. To avoid this bias the mean intensity of each image was calculated from the middle 80 % of the tonal range. To improve the visibility of the dura, the total tonal range was then expanded by compressing 10 % of the brightest and darkest voxels to their respective extremes. Further, the images were made brighter by a gamma correction with gamma set to 0.8. The gamma correction was performed using the function y = b * (x/b)^γ^ where y = output data, b = bit depth, x = input data, and γ = gamma value. Finally, the image size on the screen was scaled to 0.25 (0.5 × 0.5) times the true size of data, which meant that a 1 mm horizontal or vertical line on the screen corresponded to 2 mm in the image data.

The segmentations were performed in sagittal orientation by tracing the dural margins using the landmarks described by Eritaia et al. [4]. If the dura was not visible its location was estimated by visual extrapolation guided by the cerebral contour and surrounding dura mater. The segmentation crosses the foramen magnum at the arches of the atlas (C1), continuing along the clivus and excluding the pituitary gland by traversing the sella turcica from the dorsum sellae to the jugum sphenoidale. Further on, the inferior surface of the frontal lobe is followed. Using these landmarks the superior sagittal sinus, the confluence of sinuses, the transverse sinuses, the sigmoid sinuses and the occipital sinus were included. All measurements started at the slice near the longitudinal fissure where the cerebral aqueduct was most prominent and continued laterally in both directions until the last traces of the meninges disappeared. The software enables markers to be placed in coronal orientation, which then appear in sagittal orientation. In the present study such markers were used to guide the segmentation at the lateral ends of the dura. The mean number of sagittal slices was 136 per MRI scan with a standard deviation of 5 slices, and the segmentations took on average a little over two and a half hours per scan. N. Klasson performed the segmentations.

**Figure 2:**
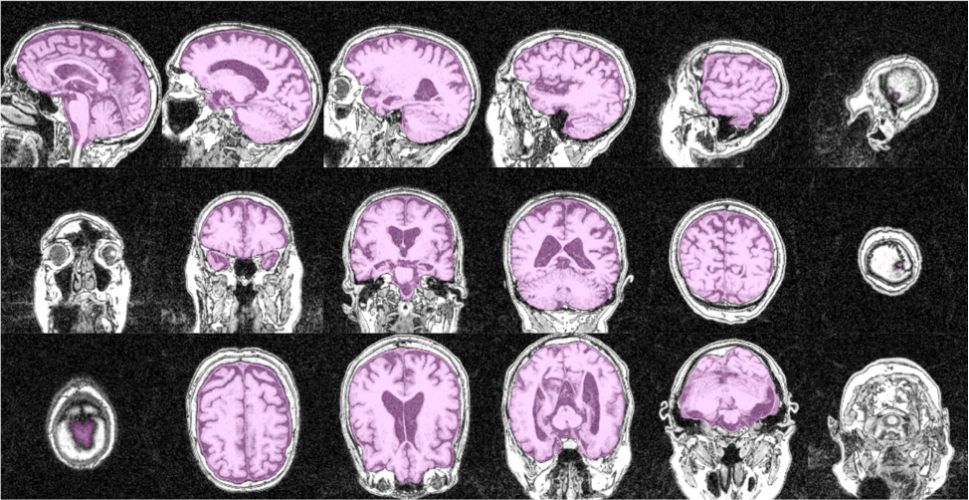
**Illustration of a segmentation.** The first row shows a sagittal segmentation from the intracranial area where the cerebral aqueduct was most prominent to one of the lateral ends of the cranial vault. The second row shows the coronal reconstruction of the same segmentation, and the third row the transversal reconstruction; both reconstructions are visualized from the first to the last slice where at least one voxel has been classified as intracranial volume.

Using the MATLAB (version R2012b) function inpolygon, each voxel within the sagittal ICAs was traced resulting in a 3D matrix of voxels describing the intracranial vault. This 3D matrix was then used to calculate ICAs in coronal and transversal orientation. That is, ICV segmentations were reconstructed as if they had been segmented in coronal and transversal orientation. This was done to evaluate how the linear spacing affects not only ICV estimates based on sagittal ICA segmentations, but also those based on coronal and transversal ICAs. An illustration of the initial sagittal segmentation and its coronal and transversal reconstructions can be viewed in Figure 2.

New sagittal segmentations were performed six months after the first ICV segmentations to enable intra-rater reliability analysis. This was done in two sessions by first re-segmenting every 10^th^ mm on 31 randomly selected MRI scans and then every 40^th^ mm on the remaining 31 scans. The image settings were the same as during the first segmentation process and the segmentations began at the sagittal slice where the cerebral aqueduct was most prominent. The segmentations took on average 13 ½ minutes when segmenting every 10^th^ mm, and 4 ½ minutes when segmenting every 40^th^ mm. Using the same procedure and group selection as in the intra-rater analysis, one additional rater (S. Skau) performed segmentations for inter-rater reliability. On average these segmentations took 22 ½ minutes when segmenting every 10^th^ mm and 5 minutes when segmenting every 40^th^ mm. Both raters were blinded to participant age, gender, and cognitive status as well as to previous segmentations during all ICV segmentations.

### Estimate calculation

ICV estimates were derived from the ICV segmentations, following and extending the procedure of Eritaia et al. [4]. Briefly, subsamples of linearly spaced ICAs were interpolated into ICV estimates with the position of the first ICA restricted to be within one linear spacing from the outermost ICA. Thus, using a linear spacing of n results in n possible subsamples of ICAs and therefore n possible estimates for each of the 62 ICV segmentations. To calculate the validity of the estimates, combinations containing one estimate for each ICV segmentation were used. Such a combination can be derived in n^62^ (n_ICV1_ * n_ICV2_ * n_ICV3_… * n_ICV62_) different ways (the multiplication principle). The validity of each of the combinations depends on which estimates were chosen, and then not only on the linear spacing, but also on which positions were used for the first ICAs. Therefore, to describe the validity of estimates only due to linear spacing, independently of the position of the first ICA, the validity of all possible combinations must be described. As it is practically impossible to evaluate all of these n^62^ combinations of estimates, 2000 combinations were chosen randomly for each linear spacing. The randomization was done by randomly choosing one estimate out of the n possible for each ICV segmentation to construct one combination, and then this procedure was repeated 2000 times.

The whole process was carried out for the sagittal, coronal and transversal ICAs separately and was done using linear spacings ranging from 2 mm up to 50 mm. The first coronal ICAs were chosen anteriorly and the first transversal ICAs superiorly.

Three different interpolation methods were evaluated using the same randomly chosen combinations of ICA subsamples for each orientation. The methods were 1) a piecewise constant interpolation, 2) a piecewise linear interpolation and 3) a cubic spline interpolation. The last two are different kinds of piecewise polynomial interpolation methods and were implemented using the MATLAB functions interp1 and spline respectively. To assure interpolation up to the sagittal borders of the intracranial vaults, zeros (indicating an ICA of zero mm^2^) were added at the positions were the intracranial vaults ended. This addition of zero ICAs is not useful for the piecewise constant interpolation.

### Statistical analysis

A total of 441 (3 interpolation methods * 3 ICA orientations * 49 linear spacings) unique estimation settings were evaluated using 2000 combinations of ICV estimates for each. For each setting, Jaccard index values, Pearson’s linear correlation coefficients, and intra-class correlation coefficients (ICC) were calculated for each of the 2000 combinations in relation to the ICV segmentations. Percentage errors for all estimates in these combinations were also calculated. The ICC calculations were performed using a two-way random effects model for single measurements and absolute agreement [15].

To evaluate if the percentage errors of the cubic spline interpolation differed significantly from those of the piecewise constant interpolation, the mean absolute percentage error of the n estimates of each ICV segmentation was calculated for each linear spacing and orientation. These mean absolute percentage errors were then compared between the two interpolation methods for each linear spacing and orientation using Student’s t-tests for paired data.

Intra- and inter-rater ICCs were calculated by comparing the re-segmentations with the corresponding subsamples of sagittal ICAs obtained from the first ICV segmentations, excluding all other ICAs. These ICC values were calculated using a two-way mixed effects model for single measurements and absolute agreement.

As a way of evaluating the ICV segmentations, associations of ICV to age, gender and group belonging (controls, GDS 2, 3 or 4) were analyzed. The possible correlation between ICV and age was evaluated using Pearson’s linear correlation coefficient, gender difference was evaluated using an independent-samples t-test, and difference due to group belonging by a Kruskal-Wallis test. As the male to female ratio differed between controls and patients, the Kruskal-Wallis test was also performed for males and females separately.

All statistics were performed in MATLAB (version R2012b) and *p* values less than or equal to 0.05 were regarded as statistically significant. Pearson’s correlations were calculated using the MATLAB function corr, t-test was performed using the function ttest, and the Kruskal-Wallis tests using the function kruskalwallis. ICC values were calculated using the external MATLAB function ICC written by Arash Salarian that is available online at the MATLAB file exchange homepage [16], and Jaccard index values by the function $\left({\displaystyle \sum_{n=1}^{62}} \min \left({x}_n,{y}_n\right)\right)/\left({\displaystyle \sum_{n=1}^{62}} \max \left({x}_n,{y}_n\right)\right)$ [17], where x is a vector containing the 62 actual ICVs and y a vector containing the given combination of corresponding ICV estimates.

## Results

**Figure 3:**
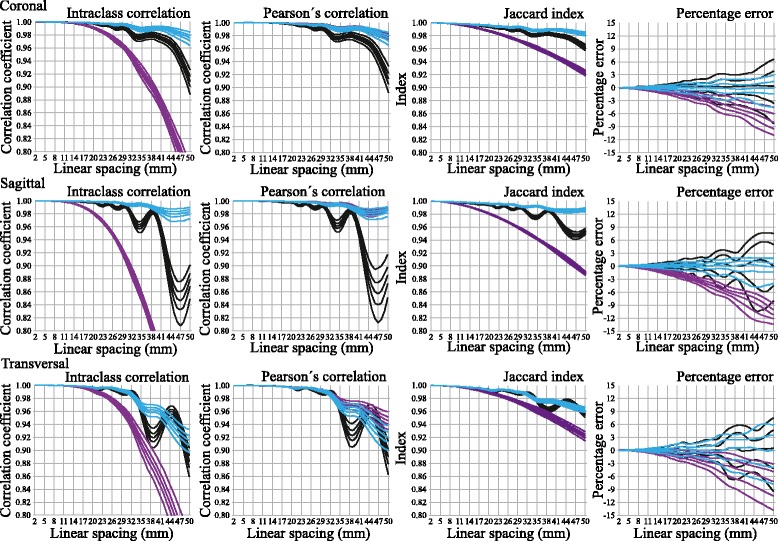
**Validity of intracranial volume estimates.** The validity of intracranial volume (ICV) estimates compared to the ICV segmentations is given by percentile curves (95^th^, 75^th^, 50^th^, 25^th^ and 5^th^ percentile) for piecewise constant interpolation (black graphs), piecewise linear interpolation (purple graphs) and cubic spline interpolation (blue graphs). The percentiles are calculated for intra-class correlation coefficients (first column), Pearson’s correlation (second column), Jaccard index (third column), and percentage errors (fourth column). Besides interpolation method the validity of the estimates depends upon the linear spacing between intracranial areas (X-axis) and orientation of the intracranial areas, either coronal (first row), sagittal (second row) or transversal (third row).

Figure 3 shows the ICCs, Pearson’s correlations, Jaccard index values, and percentage errors of the ICV estimates compared to the ICV segmentations. Except for some oscillations, particularly visible for piecewise constant interpolation, the ICCs, Pearson’s correlations, and Jaccard index values generally decreased and the percentage errors increased with increased linear spacing. Similarly, the variance of these validity measures increased with increased linear spacing regardless of interpolation method and orientation of the ICAs. Still, the decrease in validity differed depending on interpolation method and orientation of the ICAs, as seen in Figure 3.

**Figure 4:**
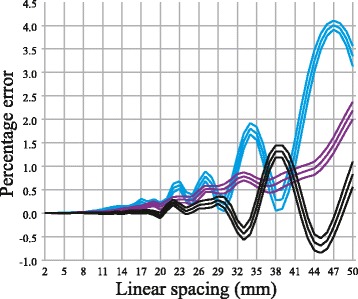
**Differences in absolute percentage error between piecewise constant and cubic spline interpolation.** T-test of the mean absolute percentage error between intracranial volume estimates calculated by piecewise constant interpolation and cubic spline interpolation using coronal (purple graphs), sagittal (blue graphs) and transversal (black graphs) intracranial areas. For each orientation the mean and 95 percent confidence intervals are illustrated. Positive percentage errors indicate the improvements when using cubic spline interpolation compared to using piecewise constant interpolation and negative percentages the opposite.

With linear spacings above or equal to 12 mm, the t-test analyses revealed that cubic spline interpolation with coronal or sagittal ICAs decreased the percentage error compared to using piecewise constant interpolation. Below 12 mm, or with the use of transversal ICAs, the difference was not always significant. Using transversal ICAs, cubic spline interpolation could even result in larger percentage errors compared to when using piecewise constant interpolation. Means and confidence intervals of the percentage error differences between the two interpolation methods are presented in Figure 4.

The ICC value for intra-rater reliability in the present study was 0.996, both when segmenting every 10^th^ and when segmenting every 40^th^ mm. The inter-rater reliability was 0.991 in the case of every 10^th^ mm and 0.987 in the case of every 40^th^ mm.

**Figure 5:**
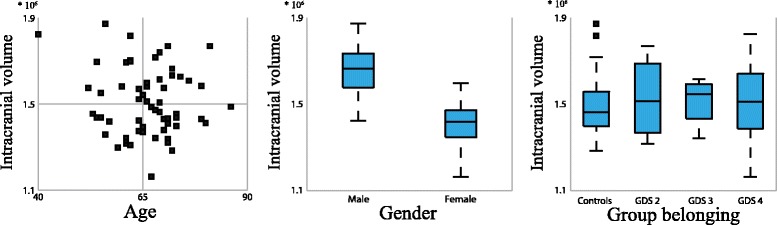
**Relation between intracranial volume and age,** **gender and group belonging.** Relation between the intracranial volumes and age, gender and group belonging. The groups consists of controls and patients with stage two, three or four on the global deterioration scale (GDS). Age is given in years and intracranial volume in cubic millimeter.

No correlation between ICV and age could be seen (*p* = 0.376). Further, the distributions of the ICVs did not differ due to group belonging (*p*_all_ = 0.977, *p*_females_ = 0.458, *p*_males_ = 0.672), while a difference in mean ICV could be seen by gender (*p* < 0.001) where the females had a mean ICV of 1416955 mm^3^ (standard deviation: 91678 mm^3^) and the males a mean ICV of 1658268 mm^3^ (standard deviation: 115535 mm^3^). In Figure 5 the ICVs are displayed in relation to age, group belonging and gender.

## Discussion

The present study shows that a high validity of ICV estimates, calculated by linearly spaced ICAs, could be maintained also at larger linear spacings if using cubic spline interpolation and coronal or sagittal ICAs rather than piecewise constant interpolation or transversal ICAs.

The first aim of the present study was to replicate a study by Eritaia et al. [4]. In that study it is described how the linear spacing of sagittal ICAs affects the validity of ICV estimates using piecewise constant interpolation. A relationship that was identical for practical purposes was found in the present study. In both studies the validity of the ICV estimates decreased and grew more uncertain with increased linear spacing. For a more detailed comparison the difference in linear spacing between the two studies must be taken into account. As Eritaia et al. used a spacing between slices (slice thickness plus slice gap) of 0.938 mm, a segmentation of every 25^th^ slice equals to a linear spacing of 23.45 mm (25^th^ slice * 0.938 mm/slice). At this linear spacing Eritaia et al. found an ICC of 0.993-0.997 between the 5^th^ and 95^th^ percentiles. In the present study the ICC values at a linear spacing of 24 mm were 0.993-0.996.

Unlike in the study by Eritaia et al. [4], percentiles of the percentage errors for each linear spacing were calculated instead of percentiles of their maximum at each combination. This was done mainly to reduce the influence of extremes that could bias the evaluation. Hence, the present results should be more generalizable. The already established use of sagittal ICAs with small linear spacing for ICV estimation when using piecewise constant interpolation was still supported by the current results.

The second aim of the present study was to extend our knowledge about how linear spacing affects the validity of ICV estimates to also include coronal and transversal ICAs. The piecewise constant interpolation method has been used both in transversal [1] and coronal orientation [18]. The present study shows that there is a difference in validity depending on the orientation of the ICAs. This difference is negligible at smaller linear spacings (<= 15 mm), but as the linear spacing increases the differences become more apparent. At larger linear spacings, coronal ICAs resulted in the most robust and valid ICV estimates when using piecewise constant interpolation, while both sagittal and transversal ICAs showed large shifts in validity. Most studies use small linear spacings and the validity in these studies should thus be high regardless of the orientation of the ICAs.

As the coronal and transversal ICAs used in the present study were reformatted from sagittally segmented volumes, possible difficulties associated with actually segmenting in coronal or transversal orientation are not covered. Further, the MRIs were not realigned to correct for head tilt. While there was barely any lateral tilt of the heads, the coronal plane had been aligned during the MRI examinations so that it was perpendicular to the longitudinal axis of hippocampus (a slight backward tilt of the head). This alignment of the coronal plane makes the results for the transversal and coronal orientations less generalizable for studies where a similar alignment is not used. The generalizability should still be high, but less so for large linear spacings in these orientations.

**Figure 6:**
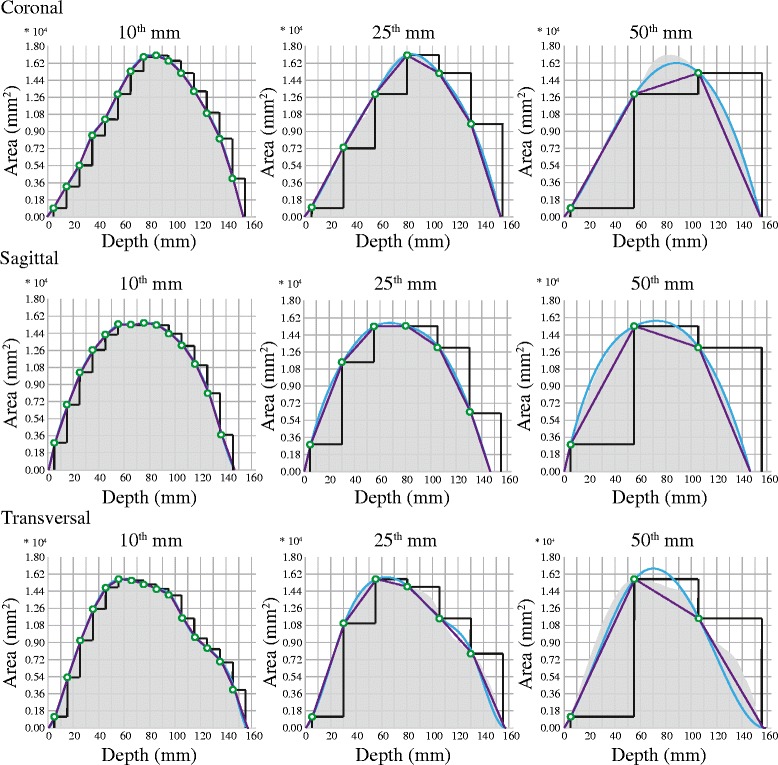
**Estimates of an intracranial volume visualized in comparison to the actual volume.** Visualization of a randomly chosen intracranial volume compared to 27 different estimates of this volume. The estimates differ with respect to interpolation method, linear spacing and orientation of the intracranial areas used to calculate the estimates. The gray areas represent the actual volume and the green dots the known intracranial areas (ICA). The estimates are visualized by black graphs (piecewise constant interpolation), purple graphs (piecewise linear interpolation) and blue graphs (cubic spline interpolation). The X-axis describes at what depths the ICAs were measured and the Y-axis the size of the areas. Coronal ICAs are ordered from anterior to posterior and transversal ICAs from superior to inferior. All estimates in the illustration were calculated beginning with the ICA at a depth of 5 mm.

The third aim of the present study was to evaluate if the validity of the ICV estimates could be improved by the use of another interpolation method than piecewise constant interpolation. Using the polynomial interpolation methods the oscillations were reduced, resulting in more robust ICV estimates. While the piecewise linear interpolation increasingly underestimated the volumes, resulting in low ICC and Jaccard index, the Pearson’s correlation remained high. This was true regardless of ICA orientation. The cubic spline interpolation on the other hand resulted in improvements of ICC, Jaccard index, Pearson’s correlation and absolute percentage error (Figure 4), but only for sagittal and coronal ICAs. It seems that because the cubic spline interpolation assumes a smoothly varying shape, it failed to consistently recover the lost information of the more irregular transversal ICAs. Figure 6 summarizes, by means of an example, how the different interpolation methods work at the different orientations and linear spacings.

In large studies manual estimation of ICV is seldom used. Two and a half hour per ICV might seem impossibly time-consuming. Even segmenting every 10^th^ mm, which takes some 15 minutes per ICV, is generally considered to be too time consuming. This is the main reason why automatic ICV estimation has been preferred in these settings. Two commonly used automatic approaches, evaluated by Nordenskjöld et al. [19], are FreeSurfer [20] and SPM [21]. Nordenskjöld et al. found that FreeSurfer (version 5.1.0) had a Pearson’s correlation of 0.937 to manual ICV segmentations while SPM 8 had a Pearson’s correlation of 0.856 (correlations calculated by the square root of R^2^ from univariate linear regression models). In a more recent study by Malone et al. FreeSurfer (version 5.3.0) had a Pearson’s correlation of 0.895 to manual estimates of ICV, while SPM 8 and SPM 12 had Pearson’s correlations of 0.760 and 0.970 respectively to such estimates [22]. Better correlations can be achieved using manual segmentations even at a linear spacing of 50 mm. In the present study the median Pearson’s correlation at this linear spacing was 0.985 using sagittal ICAs and cubic spline interpolation. The time needed for such manual segmentations should be less than 5 minutes per ICV. Possible bias due to pathological brain atrophy, as might be the case with FreeSurfer (version 3.0.2) [23], or even due to age related atrophy as seen in SPM 8 [19] would also be avoided.

Beside the commonly used FreeSurfer and SPM, there might exist automatic methods for ICV assessment that perform as well as manual segmentations of every 50th mm. For example, Keihaninejad et al. showed promising results in that two out of four evaluated automatic methods had ICCs of 0.98-0.99 to manual estimates of ICV [12]. While the evaluations were done for T1-weighted images from both 1.5 T and 3 T scanners, the comparisons for each scanner were done using manual segmentations from only five healthy participants. Like for many, if not all, automatic ICV estimation methods, more thorough evaluations are needed, including the important aspect that the methods should not be biased by atrophy.

The evaluated algorithms for ICV estimation in the present study require MRI scans that include the whole intracranial vault. This requirement might exclude participants with larger heads if care is not taken during the MRI examinations. This could be the case in the present study where the eight excluded participants had a higher proportion of males compared to the remaining participants. The generalizability of the present study could therefore be questioned. Further, as the estimates were computed from the same ICV segmentations they were compared to, the validity results in Figure 3 are only affected by variance due to linear spacing. When adding other sources of variance to the measures, such as intra-rater variance, the ICCs can be expected to drop. This was also confirmed when evaluating the intra- and inter-rater effect on the sagittal segmentations. Regardless of interpolation method, the drop in ICC for sagittally estimated ICVs just due to intra-rater variance (ICC = 0.996) was larger than that due to a linear spacing of 10 mm (5^th^ percentile of ICCs > 0.998). Thus, properties of the population to be examined and other sources of variance should always be considered when choosing a linear spacing guided by the results of the present study.

The methods and settings used for preprocessing the image data in the present study might have been suboptimal. Specifically, the reformatting of the image data was done by a linear interpolation when other interpolation methods would arguably have been better [24]. However, the error introduced by the linear interpolation is likely negligible because of the large size of the ICVs.

One of the most important aspects of measures of intracranial volume is that they should not be biased by atrophy. The ICVs in the present study were segmented following the dura mater to make sure not to include such a bias in the findings. There was no difference in ICV due to group belonging (controls, GDS 2, 3 or 4) or any correlation to age, which speaks against a possible bias in the manual segmentations. The highly similar results for the piecewise constant interpolation to those of Eritaia et al. [4], that only included normal controls, also indicate that atrophy has not affected the present results.

When reporting the use of ICV estimates calculated by linearly spaced ICAs, at least three pieces of information should be included in the specifications. These are: 1) orientation of the ICAs, 2) linear spacing, preferably in mm, and 3) interpolation method. If an interpolation method other than the piecewise constant interpolation is used, it should also be noted whether zeros have been added to the subsamples of ICAs at the positions were the intracranial vaults ended (done to assure interpolation up to the borders of the cranial vaults).

## Conclusions

The present study showed that the validity of ICV estimates by linearly spaced ICAs is barely affected by the orientation of the ICAs or the choice of interpolation method at small linear spacings (<= 15 mm). At larger linear spacings cubic spline interpolation on sagittal or reconstructed coronal ICAs resulted in the most valid estimates. Even at a linear spacing of 50 mm, requiring less than five minutes segmentation per ICV, highly valid ICV estimates could be achieved.

## Abbreviations

GDS: Global deterioration scale
ICA: Intracranial area
ICC: Intra-class correlation coefficient
ICV: Intracranial volume
IR/GR: Inversion recovery/gradient echo
MCI: Mild cognitive impairment
MRI: Magnetic resonance image
